# Dual Function of a Bee Venom Serine Protease: Prophenoloxidase-Activating Factor in Arthropods and Fibrin(ogen)olytic Enzyme in Mammals

**DOI:** 10.1371/journal.pone.0010393

**Published:** 2010-05-03

**Authors:** Young Moo Choo, Kwang Sik Lee, Hyung Joo Yoon, Bo Yeon Kim, Mi Ri Sohn, Jong Yul Roh, Yeon Ho Je, Nam Jung Kim, Iksoo Kim, Soo Dong Woo, Hung Dae Sohn, Byung Rae Jin

**Affiliations:** 1 College of Natural Resources and Life Science, Dong-A University, Busan, Korea; 2 Department of Agricultural Biology, National Academy of Agricultural Science, Suwon, Korea; 3 Department of Agricultural Biotechnology, Seoul National University, Seoul, Korea; 4 Department of Agricultural Biology, Chonnam National University, Gwangju, Korea; 5 Department of Plant Medicine, Chungbuk National University, Cheongju, Korea; CNRS - Université Aix-Marseille, France

## Abstract

Bee venom contains a variety of peptides and enzymes, including serine proteases. While the presence of serine proteases in bee venom has been demonstrated, the role of these proteins in bee venom has not been elucidated. Furthermore, there is currently no information available regarding the melanization response or the fibrin(ogen)olytic activity of bee venom serine protease, and the molecular mechanism of its action remains unknown. Here we show that bee venom serine protease (Bi-VSP) is a multifunctional enzyme. In insects, Bi-VSP acts as an arthropod prophenoloxidase (proPO)-activating factor (PPAF), thereby triggering the phenoloxidase (PO) cascade. Bi-VSP injected through the stinger induces a lethal melanization response in target insects by modulating the innate immune response. In mammals, Bi-VSP acts similarly to snake venom serine protease, which exhibits fibrin(ogen)olytic activity. Bi-VSP activates prothrombin and directly degrades fibrinogen into fibrin degradation products, defining roles for Bi-VSP as a prothrombin activator, a thrombin-like protease, and a plasmin-like protease. These findings provide a novel view of the mechanism of bee venom in which the bee venom serine protease kills target insects via a melanization strategy and exhibits fibrin(ogen)olytic activity.

## Introduction

Bee venom serves as a defensive weapon against intruders (such as animals), and it contains a variety of enzymes, peptides, and biogenic amines [1]–[4]. Bee stings can cause life-threatening allergic reactions due to an immediate hypersensitivity-induced reaction that leads to anaphylaxis [5], [6]. Two of the more commonly encountered species of bee are the honeybee (*Apis mellifera*), which is a representative beneficial insect for humans, and the bumblebee (*Bombus* spp.), which is commonly used in greenhouses and fields to pollinate crops [7]. The amount of venom released by a honeybee sting is approximately 5-times greater than that released by a bumblebee sting [8]. Unlike honeybees, however, bumblebees can sting multiple times and do not lose their stingers. Bumblebee venom appears to be highly cross-reactive with honeybee venom [1]. Additionally, bee venom has been used as a traditional medicine to treat a variety of diseases, including arthritis, rheumatism, pain, cancerous tumors, and skin diseases [4], [9]. Better knowledge of the components of bee venom would be useful for improving therapeutic treatments for allergic reactions to bee stings [1], developing an immunotherapy for bee venom hypersensitivity [6], [10], and investigating the mechanism underlying venom therapy in alternative medicine [4], [11].

Previous studies have demonstrated the presence of serine proteases in bee venom [1], [2], [12]. Bee venom serine proteases, which consist of a single serine protease domain, are considered important allergens that have significant IgE binding activity [1], [2], [10]. Compared to honeybee venom, bumblebee venom contains a larger amount of serine proteases [12].

Serine proteases are found in diverse organisms and share many biochemical and structural properties, including a conserved catalytic triad (Ser, His, and Asp) that represents the main criterion for classification of a protein as a serine protease. Serine proteases have diverse functions and play roles in digestion, the immune response, complement activation, cellular differentiation, and hemostasis [13], [14]. In snake venom, a number of serine proteases show fibrin(ogen)olytic activity that acts in hemostasis and thrombosis in mammals [15]–[18]. The fibrin(ogen)olytic activity of snake venom serine proteases, which inhibits blood coagulation in victims, serves as a mechanism to facilitate the spread of toxic components throughout the bloodstream. In arthropods, an immune challenge triggers a serine protease cascade that leads to the activation of prophenoloxidase (proPO)-activating factors (PPAFs), which are also called proPO-activating enzymes (PPAEs) or proteinases (PAPs) [19], [20]. PPAFs are activated by cleavage between their clip and serine protease domains. Once activated, PPAFs convert proPO to phenoloxidase (PO), which then catalyzes the production of quinones to form melanin.

While mounting evidence points to the serine protease component of bee venom as an important allergen [1], [3], [12], the molecular function of the bee venom serine protease, unlike other major components, has not been studied. Furthermore, snake venom serine proteases have been extensively characterized [15]–[18], but the roles of these proteins in bee venom remain unknown. Here we demonstrate that bumblebee (*Bombus ignitus*) venom serine protease (Bi-VSP) is a multifunctional enzyme that functions in a different manner in arthropods and mammals.

## Results and Discussion

### Bi-VSP Possesses a Domain Structure Similar to PPAF and Snake Venom Serine Protease

**Figure 1:**
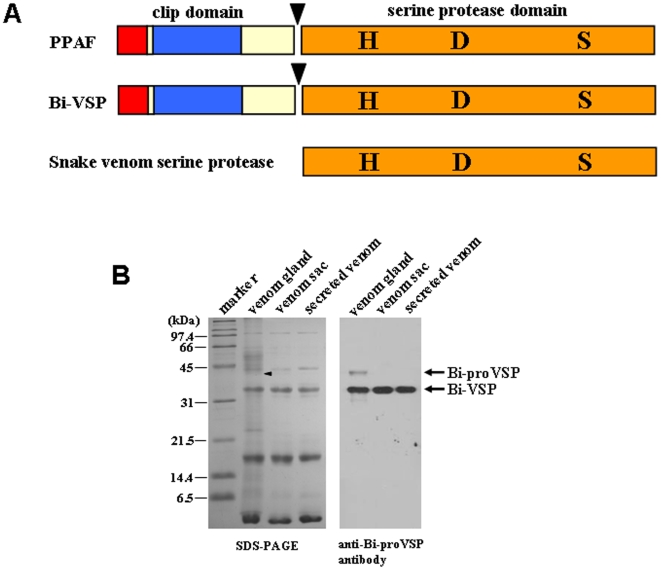
Bi-VSP is a secreted protein with a domain structure similar to those of PPAF and snake venom serine protease. (A) The domain structures of Bi-VSP, PPAF, and snake venom serine protease: hydrophobic signal sequence (red), carboxy-terminal serine protease (SP) domain (orange), single amino-terminal clip domain (blue), cleavage site (arrowhead), and residues in the catalytic triad of the SP (H, D, and S). (B) Expression of Bi-VSP. Protein obtained from the venom gland, venom sac, and secreted venom of *B. ignitus* worker bees was analyzed using SDS-PAGE (left) and western blot (right). Bi-proVSP and Bi-VSP are shown. The arrowhead on the left indicates the position of Bi-proVSP.

To explore the role of serine proteases in bee venom, we identified an expressed sequence tag (EST) for a gene encoding a venom serine protease (Bi-VSP) in the bumblebee *Bombus ignitus*. The *Bi-VSP* gene consists of six exons encoding a 360-amino acid protein (Figure S1). The predicted Bi-VSP protein contains features consistent with catalytic PPAFs, including a catalytic triad (His, Asp, and Ser) in the serine protease domain and a single clip domain (Figures 1A & S2) [20], [21]. In addition, the catalytic triad in the serine protease domain of Bi-VSP is consistent with snake venom serine proteases (Figures 1A & S3) [15], [16]. Thus, Bi-VSP possesses a catalytic domain structurally similar to those of PPAF and snake venom serine protease.

We examined the pattern of *Bi-VSP* expression to confirm that it is a component of bee venom. *Bi-VSP* exhibits venom gland-specific expression (Figure S4). An anti-Bi-proVSP antibody was generated using a recombinant Bi-VSP precursor (40-kDa Bi-proVSP) expressed in baculovirus-infected insect cells (Figure S5). The antibody was used to demonstrate the presence of Bi-VSP in the venom gland, venom sac, and in secreted venom (Figure 1B). The form of Bi-VSP detected in bee venom was the 34-kDa mature protein, which is created by cleavage of the catalytic domain of Bi-proVSP between Arg113 and Val114 (Figure S1A). This result is consistent with data characterizing other PPAFs [20], [21] and snake venom serine proteases [15], which are expressed as zymogens that are activated by proteolytic cleavage. Taken together, these data indicate that Bi-proVSP is produced in the venom gland, gets cleaved into its 34-kDa active form (mature Bi-VSP), and is then stored in the venom sac.

### Bi-VSP Acts as a PPAF That Activates a Lethal Melanization Response

**Figure 2:**
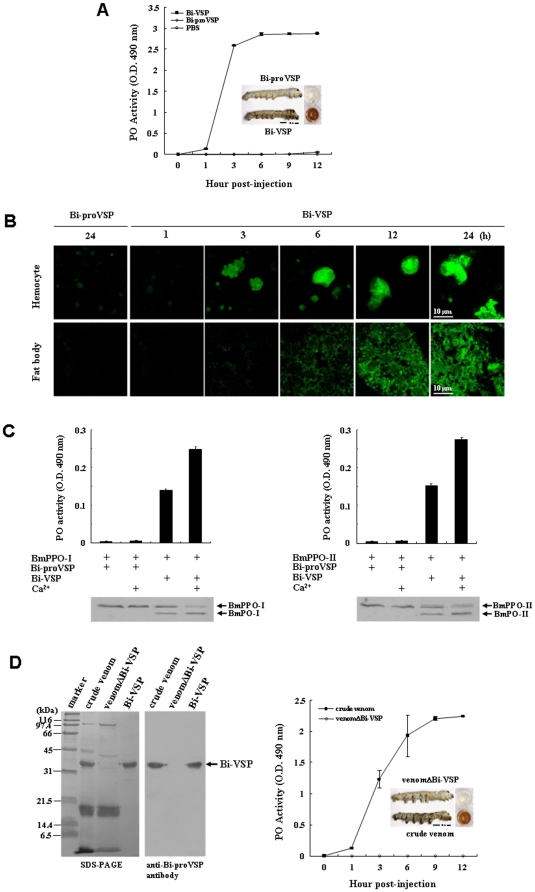
Bi-VSP acts as a PPAF. (A) PO activity in the hemolymph of fifth-instar *B. mori* larvae injected with Bi-VSP (5 µg/larva), Bi-proVSP (5 µg/larva), or phosphate-buffered saline (PBS, 30 µl/larva). PO activity is expressed as the mean ± SD (*n* = 3). (Inset) *B. mori* larvae and hemolymph 12 h p.i. (B) Immunofluorescent staining of hemocytes and fat bodies in fifth-instar *B. mori* larvae injected with Bi-proVSP (5 µg/larva) or Bi-VSP (5 µg/larva). Apoptosis (green) of hemocytes and fat bodies is visible. Bi-proVSP was used as a negative control. Scale bar, 10 µm. (C) The effects of Bi-VSP on PO activity and on the cleavage of *B. mori* proPOs. Purified BmPPO-I (left) and BmPPO-II (right) were incubated with Bi-VSP or Bi-proVSP in the presence or absence of Ca^2+^. Cleavage of the His-tagged recombinant BmPPO-I and BmPPO-II into BmPO-I and BmPO-II was detected using a western blot with an anti-His antibody. (D) Bee venom lacking Bi-VSP was unable to activate proPO cleavage and melanization. SDS-PAGE (left) and western blot (middle) showing crude venom and venom lacking Bi-VSP (venomΔBi-VSP). PO activity (right) in the hemolymph of fifth-instar *B. mori* larvae injected with crude venom or venomΔBi-VSP (5 µg/larva) is expressed as the mean ± SD (*n* = 3). (Inset) *B. mori* larvae and hemolymph 12 h p.i.

We first identified Bi-VSP as a PPAF, and we propose that it is involved in the activation of proPO and in the subsequent melanization response in target insects. Initially, we aimed to determine whether Bi-VSP is capable of activating the PO cascade in a target insect [21], and if so, whether the melanization response adversely affects the target insect [22]. To examine this issue, we selected the *Bombyx mori* silkworm as a model target insect because the proPOs in the PO cascade of this insect have been characterized [23]–[25]. Purified Bi-VSP was injected into fifth-instar *B. mori* larvae (Figure S6) and PO activity was measured (Figure 2A). The PO activity increased for 3 h in the injected larval hemolymph and continued to increase at a slower rate thereafter. No PO activity was detected upon injection of Bi-proVSP, indicating that Bi-proVSP was not cleaved into the active Bi-VSP in *B. mori* larvae. To determine whether Bi-VSP induces cell death via melanization, immunofluorescent staining of the larval cells was performed. Cell death in the hemocytes and fat bodies was significant in the Bi-VSP-treated larvae 6 h post-injection (p.i.) but was not detected in the Bi-proVSP-treated larvae (Figure 2B).

To assess whether the Bi-VSP-induced PO activity observed in *B. mori* larvae was due to cleavage of *B. mori* proPOs, recombinant *B. mori* proPO-I (BmPPO-I) and proPO-II (BmPPO-II) were produced in insect cells (Figure S7). Purified BmPPO-I and BmPPO-II were then incubated with Bi-VSP or Bi-proVSP. Further defining the role of Bi-VSP as a bee venom PPAF, Bi-VSP cleaved BmPPO-I and BmPPO-II and enhanced PO activity (Figure 2C). Although Bi-VSP cleaved *B. mori* proPOs in both the presence and absence of Ca^2+^, cleavage was more efficient in the presence of Ca^2+^, indicating that Bi-VSP is at least partially dependent on Ca^2+^ for optimal induction of PO activity [26], [27]. Given that we observed proPO activation by Bi-VSP both *in vivo* and *in vitro*, these data provide biochemical evidence that Bi-VSP is a PPAF enzyme that activates proPO in the insect proPO cascade.

We reasoned that the PO cascade would not be activated if Bi-VSP was excluded from the bee venom. A sample of bee venom lacking Bi-VSP was prepared by separating the venom components using size-exclusion chromatography. The Bi-VSP-containing fractions, which were identified using SDS-PAGE and western blot, were excluded from the remainder of the venom (Figure 2D). We then injected bee venom with or without Bi-VSP into *B. mori* larvae and measured PO activity. Bee venom lacking Bi-VSP was unable to activate proPO or induce melanization (Figure 2D). These results indicate that the PO cascade triggered by bee venom is Bi-VSP-dependent, thereby defining a specific role for Bi-VSP in bee venom.

**Figure 3:**
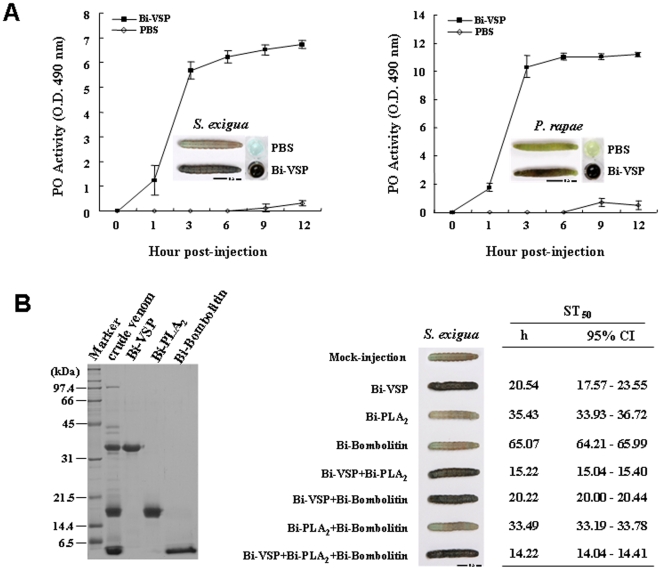
Bi-VSP activates a lethal melanization response. (A) PO activity in the hemolymph of fifth-instar *S. exigua* larvae (left) or *P. rapae* larvae (right) injected with Bi-VSP (2 µg/larva) or PBS (10 µl/larva). The PO activity is expressed as the mean ± SD (*n* = 3). (Insets) *S. exigua* larvae, *P. rapae* larvae, and hemolymph 12 h p.i. (B) Toxicity of bee venom components in *S. exigua* larvae. Bi-VSP, Bi-PLA_2_, and Bi-Bombolitin purified from *B. ignitus* venom were detected using SDS-PAGE (left). Day 2 fifth-instar *S. exigua* larvae were injected with 2 µg/larva of Bi-VSP, Bi-PLA_2_, Bi-Bombolitin, Bi-VSP + Bi-PLA_2_, Bi-VSP + Bi-Bombolitin, Bi-PLA_2_ + Bi-Bombolitin, or Bi-VSP + Bi-PLA_2_ + Bi-Bombolitin. The median survival time (ST_50_) was determined (*n* = 51), and injected *S. exigua* larvae were photographed at 24 h p.i. (right).

We also examined the Bi-VSP-mediated activation of proPO and melanization in larvae of *Spodoptera exigua* (beet armyworm) and *Pieris rapae* (Chinese cabbage worm) (Figure 3A). Both PO activity and melanization developed more rapidly and extensively in the hemolymph of *S. exigua* and *P. rapae* larvae injected with Bi-VSP than in the Bi-VSP-treated *B. mori* larvae, which may be due to differences in larval body size and hemolymph volume. The activation of a melanization response in *S. exigua*, *P. rapae,* and *B. mori* by Bi-VSP suggests that Bi-VSP may activate the PO cascade in a broad range of insects. We assessed whether Bi-VSP activation of the PO cascade plays a role in killing target insects by assaying the mortality rates of fifth-instar *S. exigua* larvae injected with 1 or 2 µg of Bi-VSP. All injected larvae exhibited melanization at 1 h p.i. (Figure S8A) and a 100% mortality within 36 h p.i. (Figure S8B). Our observations indicate that, unlike the toxic functions of other arthropod venom components identified to date [28]–[31], Bi-VSP activates a melanization response and ultimately induces the death of the target insect, demonstrating that excessive melanization due to hyperactivation of the proPO system can fatally damage an insect.

Two major components of honeybee venom are melittin and phospholipase A_2_ (PLA_2_). Melittin is the most abundant venom constituent [32] and is biologically similar to bombolitin [33]. PLA_2_ is the best-characterized toxic venom enzyme [34]. We previously identified PLA_2_ (Bi-PLA_2_) [35] and bombolitin (Bi-Bombolitin) [36] in *B. ignitus* venom. SDS-PAGE analysis revealed that Bi-Bombolitin, Bi-PLA_2_, and Bi-VSP constitute the three major components of *B. ignitus* venom (Figure 3B). To address whether Bi-VSP and other venom components act cooperatively to increase toxicity, Bi-PLA_2_ and Bi-Bombolitin were injected into *S. exigua* larvae singly or in combination with or without Bi-VSP. Among the three venom components, Bi-VSP was the most toxic to *S. exigua* larvae, followed by Bi-PLA_2_ (Figures 3B & S9). The survival time of larvae injected with Bi-PLA_2_ and Bi-Bombolitin was significantly shortened when these compounds were combined with Bi-VSP (Figure 3B), demonstrating that the increased toxicity is due to melanization. These results indicate that Bi-VSP acts synergistically with other venom components to enhance toxicity. Given that each bee venom component is required, the combination of other components and Bi-VSP that serves to elicit melanization is an efficient way to induce lethality.

In arthropods, PPAFs play a critical role in proPO activation [19], and successful parasitism requires inhibition of proPO activation by PPAF as an anti-melanization strategy [37]. Mutation of a signaling protease required for PPAF activation in the melanization cascade affects resistance and tolerance of infections in *Drosophila* [38]. However, hyperactivation of the PO cascade can induce melanization and adversely affect the insect's immune response [22]. Thus, hyperactivation of the PO cascade by injection of an active PPAF such as Bi-VSP is a melanization strategy that uses the target's own immune defense mechanisms. Based on the role of Bi-VSP and the arthropod proPO-activating system [19], we propose a pathway with a defined role for Bi-VSP in proPO activation (Figure S10). The pathway begins with injection of Bi-VSP through the stinger into the target insect. Bi-VSP then converts inactive proPO to active PO, thereby triggering the PO cascade and inducing a lethal melanization response. Thus, our results define a role for bee venom serine protease in the induction of melanization that results in the death of target insects.

### Bi-VSP Acts as a Prothrombin Activator and a Fibrin(ogen)olytic Enzyme

**Figure 4:**
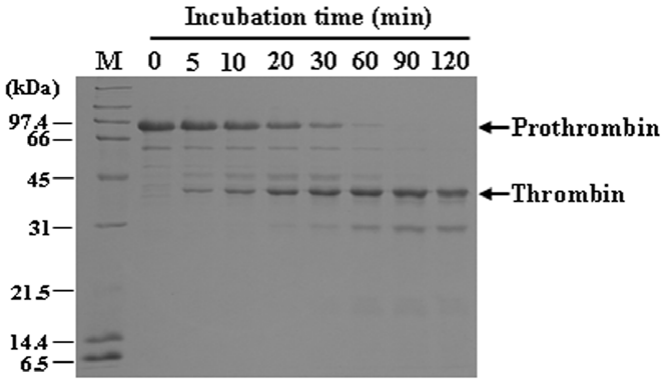
Bi-VSP activates prothrombin. SDS-PAGE analysis shows activation of human prothrombin by Bi-VSP. The number indicates the time (min) that prothrombin was incubated with Bi-VSP. Prothrombin and thrombin are shown.

Due to the fact that Bi-VSP has His, Asp, and Ser residues in positions corresponding to the catalytic triad identified in snake venom serine proteases (Figure S3), we investigated whether Bi-VSP functions in a fashion similar to snake venom serine proteases, which exhibit fibrin(ogen)olytic activity [15]–[18]. To address this issue, we analyzed the time course of human prothrombin cleavage by Bi-VSP and found that thrombin was the major cleavage product (Figure 4). This shows that Bi-VSP activates prothrombin, indicating a role for Bi-VSP as a thrombin activator. Based on the cleavage pattern observed during prothrombin activation, Bi-VSP converts prothrombin into thrombin in a manner similar to the mammalian blood coagulation factor Xa [39] (data not shown), as demonstrated for snake venom prothrombin activators [17], [40].

**Figure 5:**
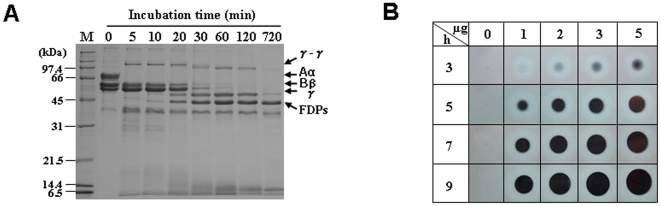
Bi-VSP possesses fibrin(ogen)olytic activity. (A) SDS-PAGE analysis of human fibrinogen hydrolysis by Bi-VSP. The number indicates the time (min) that fibrinogen was incubated with Bi-VSP. Human fibrinogen chains and fibrin degradation products (FDPs) are shown. (B) Detection of the enzymatic activity of Bi-VSP on fibrin plates. Bi-VSP (1, 2, 3, or 5 µg) was dropped onto a fibrin plate and incubated at 37°C for various periods of time.

We then assessed whether Bi-VSP possesses direct fibrin(ogen)olytic activity by analyzing the time course of human fibrinogen degradation by Bi-VSP (Figure 5A). SDS-PAGE analysis revealed that the Aα, Bβ, and γ chains of human fibrinogen disappeared within 2 h and were replaced by fibrin degradation products. To obtain direct evidence that Bi-VSP degrades fibrin, we assayed fibrinolytic activity using the fibrin plate method. This showed that addition of Bi-VSP led to the formation of a clear hollow (Figure 5B). The size of the clear hollow was dependent on the incubation time and the Bi-VSP dose. These results show that Bi-VSP not only converts fibrinogen into fibrin but also degrades fibrin into degradation products similar to those obtained using plasmin [41], [42], indicating that Bi-VSP acts as both a thrombin-like protease and a plasmin-like protease. We next addressed whether Bi-VSP is involved in the activation of plasminogen. There was no band corresponding to plasmin on SDS-PAGE, indicating that Bi-VSP is not a plasminogen activator (data not shown).

Thus, in addition to displaying PPAF activity, Bi-VSP also displays fibrin(ogen)olytic activity similar to snake venom serine proteases [15]–[18]. Our results define roles for Bi-VSP as a thrombin-like protease, as a plasmin-like protease, and as a prothrombin activator (Figure S10). This suggests that Bi-VSP affects at least three steps in the hemostatic system [15], [16], [43]. The prothrombin activation and fibrin(ogen)olytic protease activities of Bi-VSP may cooperate to effectively remove fibrinogen and thereby reduce blood viscosity [44], [45]. Our results therefore suggest that injection of the fibrin(ogen)olytic enzyme Bi-VSP is used to facilitate the spread of bee venom components throughout the bloodstream in mammals, as has been demonstrated for snake venom fibrin(ogen)olytic enzymes, which alter blood rheology by decreasing the concentration of blood fibrinogen [18], [44], [45]. Our finding that bee venom serine protease possesses fibrin(ogen)olytic activity has significant implications for the use of bee venom as a potential clinical agent in the medical and pharmacological fields of hemostasis and thrombosis [11], [18].

### Conclusion

In this present study, we provide the first evidence that bee venom serine protease functions analogously to both an arthropod PPAF and a snake venom serine protease. Furthermore, our findings highlight a rationale for the presence of a serine protease in bee venom: Bi-VSP acts as a PPAF to induce a lethal melanization response in arthropods and as a fibrin(ogen)olytic enzyme to facilitate the spread of bee venom components throughout the bloodstream in mammals. The finding that bee venom serine protease functions with a different mechanism of action in arthropods and mammals highlights the two-pronged strategy possible with bee venom serine protease.

## Materials and Methods

### Insects

The *B. ignitus*, *B. mori*, *S. exigua*, and *P. rapae* used in this study were supplied by the Department of Agricultural Biology, National Academy of Agricultural Science, Republic of Korea. *B. ignitus*, *B. mori*, and *S. exigua* were reared as previously described [46]–[48]. *P. rapae* was reared on fresh Chinese cabbage leaves at 25°C under conditions of 65±5% relative humidity using a 12 h light:12 h dark photoperiod.

### Sequence analysis and cloning

A clone encoding the *Bi-VSP* insert was selected from ESTs generated from a cDNA library created using venom glands from *B. ignitus* worker bees [35]. Plasmid DNA was extracted using the Wizard Mini-Prep Kit (Promega) and sequenced using an ABI 310 automated DNA sequencer (Perkin-Elmer Applied Biosystems). Sequences were compared using the DNASIS and BLAST programs (http://www.ncbi.nlm.nih.gov/BLAST). Genomic DNA was extracted from the fat body tissue of *B. ignitus* workers using the Wizard Genomic DNA Purification Kit (Promega); this was then used as a template for PCR. The sequences of the oligonucleotide primers used for amplification were forward 1 (1–24), 5′-ATGACGGGCTCCAAGATGCTGTTC-3′ and reverse 1 (363–340), 5′-TACAGCTGGCTTACCACCGACCAC-3′; forward 2 (340–363), 5′-GTGGTCGGTGGTAAGCCAGCTGTA-3′ and reverse 2 (1083–1060), TTATTGCATCGCTGGGAGAATAAA-3′. The amplification primers were designed using *Bi-VSP* cDNA sequences. All PCR products were verified by DNA sequence analysis.

### Tissue collection

*B. ignitus* worker bees were dissected on ice using a stereo microscope (Zeiss, Jena, Germany). Tissue samples (fat body, venom gland, and venom sac) were collected and washed with PBS (140 mM NaCl, 27 mM KCl, 8 mM Na_2_HPO_4_, 1.5 mM KH_2_PO_4_, pH 7.4). Hemolymph was collected in cold test tubes by puncturing the body of *B. ignitus* worker bees or by cutting the legs of *B. mori*, *S. exigua,* and *P. rapae* larvae. The hemolymph was centrifuged at 10,000×*g* for 10 min to remove the hemocytes and cell debris. The collected tissue samples were used without further processing.

### RNA extraction and northern blot

Total RNA was isolated from the fat body, midgut, muscle, venom gland, and venom sac using a Total RNA Extraction Kit (Promega). The total harvested RNA (5 µg/lane) was separated in a 1.0% formaldehyde agarose gel, transferred onto a nylon blotting membrane (Schleicher & Schuell, Dassel, Germany), and hybridized at 42°C with a probe in hybridization buffer containing 5× SSC, 5× Denhardt's solution, 0.5% SDS, and 100 µg/ml of denatured salmon sperm DNA. The *Bi-VSP* cDNA was labeled with [α-^32^P]dCTP (Amersham Biosciences) using the Prime-It II Random Primer Labeling Kit (Stratagene), and the labeled cDNA was used as a probe for hybridization. After hybridization, the membrane filter was washed three times for 30 minutes each in 0.1% SDS and 0.2× SSC at 65°C and then exposed to autoradiography film.

### Protein expression

A baculovirus/Sf9 insect cell expression system [49] was used for the production of recombinant Bi-proVSP proteins. A *Bi-proVSP* cDNA fragment containing the full-length open reading frame (ORF) was inserted into the pBAC1 vector (Clontech) to generate expression vectors that drive the expression of the recombinant protein under the control of the *Autographa californica* nucleopolyhedrovirus (AcNPV) polyhedrin promoter. Recombinant baculoviruses were propagated in Sf9 cells cultured in TC100 medium (Gibco BRL) at 27°C. Recombinant proteins were purified using a HisTrap column (Amersham Biosciences) and injected into BALB/c mice to produce a polyclonal antibody [50]. The protein concentration was determined using the Bio-Rad Protein Assay Kit. The protein samples were mixed with sample buffer, boiled for 5 min, and separated using 12% or 14% SDS-PAGE. Western blots were performed as described previously [50] using an Enhanced Chemiluminescence Western Blotting Detection System (Amersham Biosciences).

### Venom component purification and N-terminal sequencing

The complete sting apparatus was removed from each of ∼800 worker bees after anesthesia was induced by chilling. Venom sacs were cut free and immersed in PBS and the supernatant was collected after centrifugation at 10,000×*g* for 10 min. Bi-VSP, Bi-PLA_2_, and Bi-Bombolitin were purified from the supernatant by size-exclusion column chromatography. Size-exclusion chromatography was performed at a flow rate of 0.5 ml/min using a HiPrep 16/60 Sephacryl S-100 column (Amersham Biosciences) equilibrated with 0.05 M sodium phosphate buffer (pH 7.2) containing 0.15 M NaCl. Purified Bi-VSP, Bi-PLA_2_, and Bi-Bombolitin were identified by SDS-PAGE. To prepare bee venom lacking Bi-VSP, venom components were separated using size-exclusion chromatography as described above. The Bi-VSP-containing fractions, which were identified using SDS-PAGE and western blot, were excluded from the remainder of the venom. The Bi-VSP-lacking fractions were concentrated using SpeedVac centrifugation. The purified proteins were transferred to a polyvinylidene difluoride membrane (Applied Biosystems), stained, excised, and subjected to automated Edman degradation using a protein sequencer (Procise 491 HT Protein sequencer, Applied Biosystems).

### Experimental injections

The larvae were injected with sample solution between the sixth and seventh abdominal segments using a sterile needle. Prior to injection, the larvae were placed on ice for 5 min. After the injection, the injected site on each larva was covered with paraffin [47].

### Immunofluorescent staining

Day 2 fifth-instar *B. mori* larvae were injected with the 40-kDa Bi-proVSP or the 34-kDa Bi-VSP. Fat body tissues and hemocytes were collected at 1, 3, 6, 12, and 24 h p.i. Fat body tissues were fixed in 4% neutral buffered paraformaldehyde (NBP) for 12 h and cryoprotected by immersion in 20% sucrose phosphate buffer at 4°C for 24 h. The tissues were frozen using an optimal cutting temperature compound (Sakura Finetechnical Co., Japan), after which 10 µm sections were cut. The sections were thaw-mounted on slides at room temperature and stored at −70°C until use. Hemocytes were isolated from the hemolymph by centrifugation at 200×*g* for 10 min at 4°C prior to air-drying and fixing the samples in 4% NBP on glass slides. Apoptosis was assayed using the terminal deoxynucleotidyl transferase-mediated dUTP nick end labeling (TUNEL) technique in fat body tissues and hemocytes according to the protocol provided with the *in situ* cell death detection kit (Roche Applied Science). The samples were incubated in a TUNEL reaction mixture containing terminal deoxynucleotidyl transferase and fluorescein-conjugated dUTP at 37°C for 1 h. Apoptosis in the tissues was analyzed by laser scanning confocal microscopy (Carl Zeiss LSM 510, Zeiss).

### PO activity assay in hemolymph

The assay for PO activity in hemolymph was carried out as previously described [51]. Hemolymph from fifth-instar B. mori, S. exigua, and P. rapae larvae injected with the compounds was collected individually at various time points. To obtain cell-free hemolymph, the samples were centrifuged at 800×g for 5 min at 4°C. Optical density measurements were obtained at 490 nm. Three independent measurements of PO activity were performed. The mean values with their respective error bars are shown.

### proPO cleavage and activity assays

*B. mori* PPO-I (*BmPPO-I*) and PPO-II (*BmPPO-II*) cDNAs were reverse transcribed using the following primer sets: 5′-GGATCCATGCTGACGCCAAGAACAAC-3′ (forward) and 5′-CTCGAGCCCCTGCTGGCCGCGCTGGCG-3′ (reverse) for *BmPPO-I* (GenBank accession no. NM_001043870) and 5′-GGATCCATGGCTGACGTTTTTGAAAGC-3′ (forward) and 5′-GCGGCCGCAACTGACATGGGAGGGTTCCG-3′ (reverse) for *BmPPO-II* (GenBank accession no. NM_001112747). *BmPPO-I* and *BmPPO-II* cDNA fragments containing the full-length ORFs were inserted into the pBAC1 vector (Clontech) to express the recombinant protein under the control of the AcNPV polyhedrin promoter. Recombinant baculoviruses were propagated in Sf9 cells cultured in TC100 medium (Gibco BRL) at 27°C. The recombinant proteins were purified using a HisTrap column (Amersham Biosciences). The effect of 34-kDa Bi-VSP and 40-kDa Bi-proVSP on the cleavage of *B. mori* proPOs was examined using purified components. BmPPO-I (1 µg) or BmPPO-II (1 µg) and purified Bi-VSP (0.5 µg) or Bi-proVSP (0.5 µg) were added to 20 µl of aqueous buffer (20 mM Tris-HCl, pH 8.0) in the presence or absence of 5 mM CaCl_2_. Samples were incubated at 30°C for 10 minutes and analyzed by western blot with an anti-His antibody (Clontech). Samples were also added to 980 µl of substrate solution (20 mM L-dopamine in 20 mM Tris-HCl buffer, pH 8.0), and PO activity was measured at 490 nm [51].

### Toxicity assay

To compare the toxicity of the venom components, day 2 fifth-instar *S. exigua* larvae were injected with venom components individually and in combination. Larvae were injected with 2 µg/larva of Bi-PLA_2_, Bi-Bombolitin, Bi-VSP, Bi-VSP + Bi-PLA_2_, Bi-VSP + Bi-Bombolitin, Bi-PLA_2_ + Bi-Bombolitin, or Bi-VSP + Bi-PLA_2_ + Bi-Bombolitin. The accumulated mortality was measured for 72 h p.i. The median survival time (ST_50_) was calculated using the Probit method and the SoftTOX software, version 1.1 (WindowChem Inc., USA). The 95% confidence intervals (CI) were also calculated for the ST_50_ values.

### Prothrombin activation assay

A prothrombin activation assay was performed as previously described [52], [53]. Human prothrombin (2 µg, Sigma) was incubated with 2 ng of Bi-VSP in 50 mM Tris-HCl buffer (pH 8.0) containing 100 mM NaCl and 5 mM CaCl_2_ at 37°C for various periods of time. Aliquots were removed at various time points and analyzed using 14% SDS-PAGE.

### Fibrinogen cleavage assay

A fibrinogen cleavage assay was performed as previously described [54]. Human fibrinogen (10 µg, MP Biomedicals) was incubated with 0.25 µg of Bi-VSP in 50 mM Tris-HCl buffer (pH 8.0) at 37°C for various lengths of time. Aliquots were removed at various time points and analyzed using 14% SDS-PAGE.

### Fibrin plate assay

The fibrin plate assay was performed with 5 ml human fibrinogen (0.5%) clotted with three units of thrombin (Sigma). Bi-VSP of various concentrations was dropped onto the fibrin plate and incubated at 37°C for various periods of time. Fibrinolytic activity was determined by examining the formation of a clear hollow [55].

### Statistical analysis

Data are expressed as the mean ± SD of experiments performed in triplicate and were analyzed for statistical significance using a one-way analysis of variance (ANOVA). Differences with a *P-*value less than 0.05 were considered statistically significant for all treatments.

## Supporting Information

Figure S1 Predicted amino acid sequence and structure of Bi-VSP. (A) The deduced amino acid sequence of Bi-VSP (GenBank accession no. FJ159443). The cleavage site for the predicted signal sequence (open triangle) is indicated. Cleavage of the catalytic serine protease domain between Arg113 and Val114 (solid triangle) was confirmed by N-terminal amino acid sequencing. Conserved cysteine residues in the clip domain and serine protease (SP) domain are marked with boxes. Residues in the catalytic triad of the SP domain [His (H), Asp (D), and Ser (S)] are indicated by circles. (B) The genomic structure of the Bi-VSP gene (GenBank accession no. FJ159442) was inferred from an analysis of the Bi-proVSP cDNA. The numbers indicate the position in the genomic sequence.
(1.05 MB TIF)

Figure S2 Amino acid sequence alignment of Bi-VSP with known PPAFs. Identical residues are shown in solid boxes. The dashes represent gaps that were introduced to preserve the alignment. The conserved cysteine residues in the SP domain are marked (solid squares), and the residues in the catalytic triad of the SP domain [His (H), Asp (D), and Ser (S)] are indicated with asterisks. The abbreviations and GenBank accession numbers for the aligned sequences are: Bi-VSP (this study, FJ159443); HdPPAF-III, *Holotrichia diomphalia* PPAF-III (BAC15604); MsPAP-3, *Manduca sexta* PAP-3 (AAX18637); Dmeaster, *D. melanogaster* easter (NP_524362); HdPPAE-I, *H. diomphalia* PPAE-I (BAA34642); MsPAP-1, *M. sexta* PAP-1 (AAX18636); and BmPPAE, *B. mori* PPAE (NP_001036832). The Bi-VSP sequence was used as a reference for the identity/similarity (Id/Si) values.
(3.79 MB TIF)

Figure S3 Amino acid sequence alignment of Bi-VSP with known snake venom serine proteases. Identical residues are shown in solid boxes. The dashes represent gaps that were introduced to preserve the alignment. The conserved cysteine residues in the SP domain are marked (solid squares), and the residues in the catalytic triad of the SP domain [His (H), Asp (D), and Ser (S)] are indicated with asterisks. The abbreviations and GenBank accession numbers for the aligned sequences are: Bi-VSP (this study, FJ159443), Oscutarin C (AY940204), Batroxobin (AAA48553), TSV-PA (Q91516), PA-BJ (P81824), Halystase (P81176), and RVV-V (P18964). The Bi-VSP sequence was used as a reference for the identity/similarity (Id/Si) values.
(3.74 MB TIF)

Figure S4 Northern blot analysis of Bi-VSP. (A) A northern blot for Bi-VSP was performed using total RNA isolated from the fat bodies, midgut, muscle, venom glands, and venom sacs of *B. ignitus* worker bees. (Top) Ethidium bromide staining of the RNA is shown to indicate equal loading. (Bottom) Bi-VSP transcripts. (B) A northern blot for Bi-VSP was performed using total RNA isolated from whole bodies of worker, male, and queen bees. (Top) Ethidium bromide staining of the RNA is shown to indicate equal loading. (Bottom) The Bi-VSP signal was present as a single band for the worker and queen, but not for the male, which lacks a sting apparatus.
(0.68 MB TIF)

Figure S5 Purified recombinant Bi-proVSP. SDS-PAGE (left) and western blot (right) of recombinant Bi-proVSP purified from baculovirus-infected insect cells. The anti-Bi-proVSP antibody was produced in mice injected with recombinant Bi-proVSP.
(0.64 MB TIF)

Figure S6 Viability of *B. mori* larvae injected with Bi-VSP. The viability of day 2 fifth-instar *B. mori* larvae injected with 1-10 µg of Bi-VSP per larva was surveyed at 24 h p.i. The data are expressed as the mean ± SD of assays performed in triplicate (n = 33).
(0.62 MB TIF)

Figure S7 Purified recombinant *B. mori* proPOs expressed in baculovirus-infected insect Sf9 cells. SDS-PAGE (left) and western blot (right) of purified recombinant *B. mori* proPO-I (BmPPO-I) and proPO-II (BmPPO-II). His-tagged recombinant BmPPO-I and BmPPO-II were identified using an anti-His antibody. Lane 1, purified BmPPO-I; lane 2, purified BmPPO-II.
(0.66 MB TIF)

Figure S8 The mortality of *S. exigua* larvae injected with Bi-VSP. Day 2 fifth-instar *S. exigua* larvae were injected with Bi-VSP (1 µg or 2 µg per larva). (A) Fifth-instar *S. exigua* larvae and the collected hemolymph were photographed at various time points p.i. Scale bar, 0.5 cm. (B) The accumulated mortality was surveyed at various time points p.i. The data are expressed as the mean ± SD of assays performed in triplicate (n = 33).
(1.09 MB TIF)

Figure S9 Mortality of Bi-VSP, Bi-PLA2, and Bi-Bombolitin in *S. exigua* larvae. Day 2 fifth-instar *S. exigua* larvae were injected with 2 µg/larva of Bi-VSP, Bi-PLA2, Bi-Bombolitin, Bi-VSP + Bi-PLA2, Bi-PAP + Bi-Bombolitin, Bi-PLA2 + Bi-Bombolitin, or Bi-VSP + Bi-PLA2 + Bi-Bombolitin. The data are expressed as the mean ± SD of three assays (n = 51).
(0.78 MB TIF)

Figure S10 Proposed pathway for PO activation and the fibrin(ogen)olytic nature of Bi-VSP. The injection of bee venom via the sting apparatus results in the activation of proPO via Bi-VSP in the target insect. Elevated PO activity induces the death of target insects via melanization. In mammals, Bi-VSP not only activates prothrombin, it also degrades fibrinogen into fibrin degradation products (FDPs).
(1.53 MB TIF)

## References

[1] Hoffman DR, Jacobson RS (1996). Allergens in Hymenoptera venom XXVII: Bumblebee venom allergy and allergens.. J Allergy Clin Immunol.

[2] Winningham KM, Fitch CD, Schmidt M, Hoffman DR (2004). *Hymenoptera* venom protease allergens.. J Allergy Clin Immunol.

[3] Hoffman DR (2006). Hymenoptera venom allergens.. Clin Rev Allergy Immunol.

[4] Son DJ, Lee JW, Lee YH, Song HS, Lee CK (2007). Therapeutic application of anti-arthritis, pain-releasing, and anti-cancer effects of bee venom and its constituent compounds.. Pharmacol Ther.

[5] Fitzgerald KT, Flood AA (2006). Hymenoptera stings.. Clin Tech Small Anim Pract.

[6] Golden DBK (2007). Insect sting anaphylaxis.. Immunol Allergy Clin North Am.

[7] Velthuis HHW, van Doorn A (2006). A century of advances in bumblebee domestication and the economic and environmental aspects of its commercialization for pollination.. Apidologie.

[8] Hoffman DR, Jacobson RS (1984). Allergens in Hymeoptera venom: XII – how much protein is in a sting?. Ann Allergy.

[9] Hider RC (1988). Honeybee venom: a rich source of pharmacologically active peptides.. Endeavour.

[10] Golden DBK, Kelly D, Hamilton RG, Craig TJ (2009). Venom immunotherapy reduces large local reactions to insect stings.. J Allergy Clin Immunol.

[11] Mirshafiey A (2007). Venom therapy in multiple sclerosis.. Neuropharmacology.

[12] Hoffman DR, El-Choufani SE, Smith MM, de Groot H (2001). Occupational allergy to bumblebees: Allergens of *Bombus terrestris*.. J Allergy Clin Immunol.

[13] Neurath H (1984). Evolution of proteolytic enzymes.. Science.

[14] Krem MM, Cera ED (2002). Evolution of enzyme cascades from embryonic development to blood coagulation.. Trends Biochem Sci.

[15] Braud S, Bon C, Wisner A (2000). Snake venom proteins acting on hemostasis.. Biochimie.

[16] Matsui T, Fujimura Y, Titani K (2000). Snake venom proteases affecting hemostasis and thrombisis.. Biochim Biophys Acta.

[17] Kini RM (2005). Serine proteases affecting blood coagulation and fibrinolysis from snake venoms.. Pathophysiol Haemost Thrombo.

[18] Swenson S, Markland FS (2005). Snake venom fibrin(ogen)olytic enzymes.. Toxicon.

[19] Cerenius L, Söderhäll K (2004). The prophenoloxidase-activating system in invertebrates.. Immunol Rev.

[20] Jiang H, Kanost MR (2000). The clip-domain family of serine proteinases in arthropods.. Insect Biochem Mol Biol.

[21] Piao S, Song YL, Kim JH, Park SY, Park JW (2005). Crystal structure of a clip-domain serine protease and functional roles of the clip domains.. EMBO J.

[22] Zhao M, Söderhäll K, Park JW, Ma YG, Osaki T (2005). A novel 43-kDa protein as a negative regulatory component of phenoloxidase-induced melanin synthesis.. J Biol Chem.

[23] Kawabata T, yasuhara Y, Ochiai M, Matsuura S, Ashida M (1995). Molecular cloning of insect pro-phenol oxidase: A copper-containing protein homologous to arthropod hemocyanin.. Proc Natl Acad Sci U S A.

[24] Satoh D, Horii A, Ochiai M, Ashida M (1999). Prophenoloxidae-activating enzyme of the silkworm, *Bombyx mori*. Purification, characterization, and cDNA cloning.. J Biol Chem.

[25] Asano T, Ashida M (2001). Cuticular pro-phenoloxidase of the silkworm, *Bombyx mori*. Purification and demonstration of its transport from hemolymph.. J Biol Chem.

[26] Ashida M, Ishizaki Y, Iwahana H (1983). Activation of prophenoloxidase by bacterial cell walls or beta-1,3-glucans in plasma of the silkworm, *Bombyx mori*.. Biochem Biophys Res Commun.

[27] Ashida M, Kinoshita K, Brey PT (1990). Studies on prophenoloxidase activation in the mosquito *Aedes aegypti* L. Eur J Biochem.

[28] Ownby CL, Powell JR, Jiang MS, Fletcher JE (1997). Melittin and phospholipase A2 from bee (*Apis mellifera*) venom cause necrosis of murine skeletal muscle in vivo.. Toxicon.

[29] Inceoglu B, Lango J, Jing J, Chen L, Doymaz F (2003). One scorpion, two venoms: Prevention of *Parabuthus transvaalicus* acts as an alternative type of venom with distinct mechanism of action.. Proc Natl Acad Sci U S A.

[30] Siemens J, Zhou S, Piskorowski R, Nikai T, Lumpkin EA (2006). Spider toxins activate the capsaicin receptor to produce inflammatory pain.. Nature.

[31] Metz M, Piliponsky AM, Chen CC, Lammel V, Åbrink M (2006). Mast cells can enhance resistance to snake and honeybee venoms.. Science.

[32] Gauldie J, Hanson JM, Rumjanek FD, Shipolini RA, Vernon CA (1976). Peptide components of bee venom.. Eur J Biochem.

[33] Argiolas A, Pisano JJ (1985). Bombolitins, a new class of mast cell degranulating peptides from the venom of the bumblebee *Megabombus pennsylvanicus*.. J Biol Chem.

[34] Six DA, Dennis EA (2000). The expanding superfamily of phospholipase A2 enzymes: classification and characterization.. Biochim Biophys Acta.

[35] Xin Y, Choo YM, Hu Z, Lee KS, Yoon HJ (2009). Molecular cloning and characterization of a venom phospholipase A_2_ from the bumblebee *Bombus ignitus*.. Comp Biochem Physiol B.

[36] Choo YM, Lee KS, Yoon HJ, Je YH, Woo SD (2010). Molecular cloning and antimicrobial activity of bombolitin, a component of bumblebee *Bombus ignites* venom.. Comp Biochem Physiol B.

[37] Beck MH, Strand MR (2007). A novel polydnavirus protein inhibits the insect prophenoloxidase activation pathway.. Proc Natl Acad Sci U S A.

[38] Ayres JS, Schneider DS (2008). A signaling protease required for melanization in Drosophila affects resistance and tolerance of infections.. PLoS Biol.

[39] Kawabata S, Miura T, Morita T, Kato H, Fujikawa K (1988). Highly sensitive peptide-4-methylcoumaryl-7-amide substrates for blood-clotting protease and trypsin.. Eur J Biochem.

[40] Joseph JS, Kini RM (2001). Snake venom prothrombin activators homologous to blood coagulation factor Xa.. Haemostasis.

[41] Datta G, Dong A, Witt J, Tu AT (1995). Biochemical characterization of basilase, a fibrinolytic enzyme from *Crotalus basiliscus basiliscus*.. Arch Biochem Biophys.

[42] Zhang Y, Wisner A, Xiong Y, Bon C (1995). A novel plasminogen activator from snake venom. Purification, characterization and molecular cloning.. J Biol Chem.

[43] Rijken DC, Lijnen HR (2009). New insights into the molecular mechanisms of the fibrinolytic system.. J Thromb Haemost.

[44] Koh YS, Chung KH, Kim DS (2001). Biochemical characterization of a thrombin-like enzyme and a fibrinolytic serine protease from snake (*Agkistrodon saxatilis*) venom.. Toxicon.

[45] He J, Chen S, Gu J (2007). Identification and characterization of Harobin, a novel fibrino(geno)lytic serine protease from a sea snake (*Lapemis hardwickii*).. FEBS Lett.

[46] Yoon HJ, Kim SE, Kim YS (2002). Temperature and humidity favorable for colony development of the indoor-reared bumblebee, *Bombus ignitus*.. Appl Entomol Zool.

[47] Gui ZZ, Lee KS, Kim BY, Choi YS, Wei YD (2006). Functional role of aspartic proteinase cathepsin D in insect metamorphosis.. BMC Dev Biol.

[48] Goh HG, Lee SG, Lee BP, Choi KM, Kim JH (1990). Simple mass-rearing of beet armyworm, *Spodoptera exigua* (Hübner) (Lepidoptera: Noctuidae), on an artificial diet.. Korean J Appl Entomol.

[49] Je YH, Chang JH, Kim MH, Roh JY, Jin BR (2001). A defective viral genome maintained in *Escherichia coli* for the generation of baculovirus expression vectors.. Biotechnol Lett.

[50] Choo YM, Lee BH, Lee KS, Kim BY, Li J (2008). *Pr-lynx1*, a modulator of nicotinic acetylcholine receptors in the insect.. Mol Cell Neurosci.

[51] Ligoxygakis P, Pelte N, Ji C, Leclerc V, Duvic B (2002). A serpin mutant links Toll activation to melanization in the host defense of *Drosophila*.. EMBO J.

[52] Speijer H, Govers-Riemslag JWP, Zwaal RFA, Rosing J (1986). Prothrombin activation by an activator from the venom of *Oxyuranus scutellatus* (Taipan snake).. J Biol Chem.

[53] Silva MB, Schattner M, Ramos CRR, Junqueira-de-Azevedo ILM, guarnieri MC (2003). A prothrombin activator from *Bothrops erythromelas* (jararaca-da-seca) snake venom: characterization and molecular cloning.. Biochem J.

[54] Matsui T, Sakurai Y, Fujimura Y, Hayashi I, Ohishi S (1998). Purification and amino acid sequence of halystase from snake venom of *Agkistrodon halys blomhoffii*, a serine protease that cleaves specifically fibrinogen and kininogen.. Eur J Biochem.

[55] Astrup T, Mullertz S (1952). The fibrin plate method for the estimation of fibrinolytic activity.. Arch Biochem Biophys.

