# Supplementary figures and images for: Dual Function of a Bee Venom Serine Protease: Prophenoloxidase-Activating Factor in Arthropods and Fibrin(ogen)olytic Enzyme in Mammals
Source: PLoS One. 2010 May 3;5(5):e10393. doi: 10.1371/journal.pone.0010393 (PMC2862700; doi:10.1371/journal.pone.0010393)

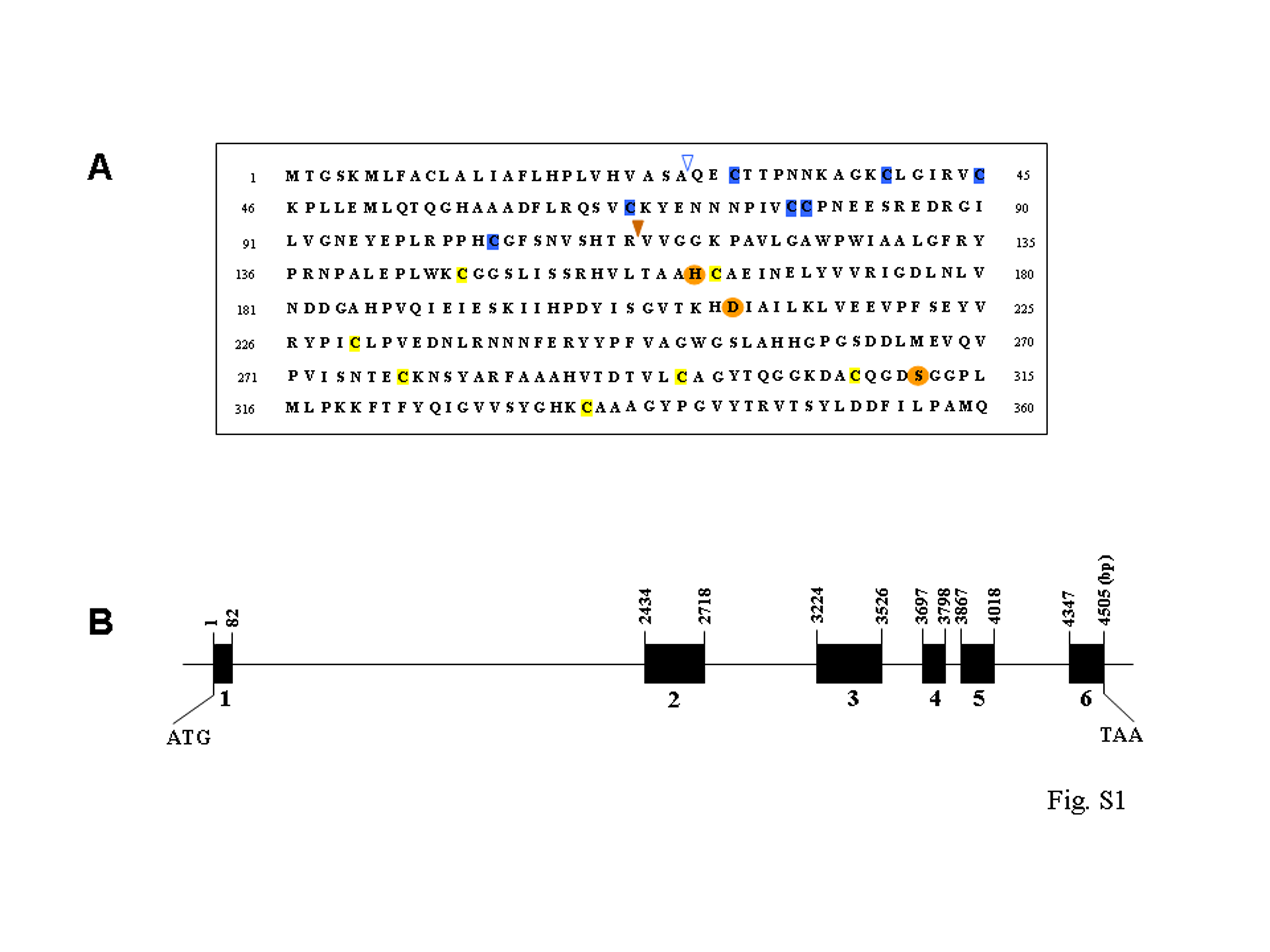

Supplement: Figure S1 — Predicted amino acid sequence and structure of Bi-VSP. (A) The deduced amino acid sequence of Bi-VSP (GenBank accession no. FJ159443). The cleavage site for the predicted signal sequence (open triangle) is indicated. Cleavage of the catalytic serine protease domain between Arg113 and Val114 (solid triangle) was confirmed by N-terminal amino acid sequencing. Conserved cysteine residues in the clip domain and serine protease (SP) domain are marked with boxes. Residues in the catalytic triad of the SP domain [His (H), Asp (D), and Ser (S)] are indicated by circles. (B) The genomic structure of the Bi-VSP gene (GenBank accession no. FJ159442) was inferred from an analysis of the Bi-proVSP cDNA. The numbers indicate the position in the genomic sequence. (1.05 MB TIF) [file pone.0010393.s001.tif]

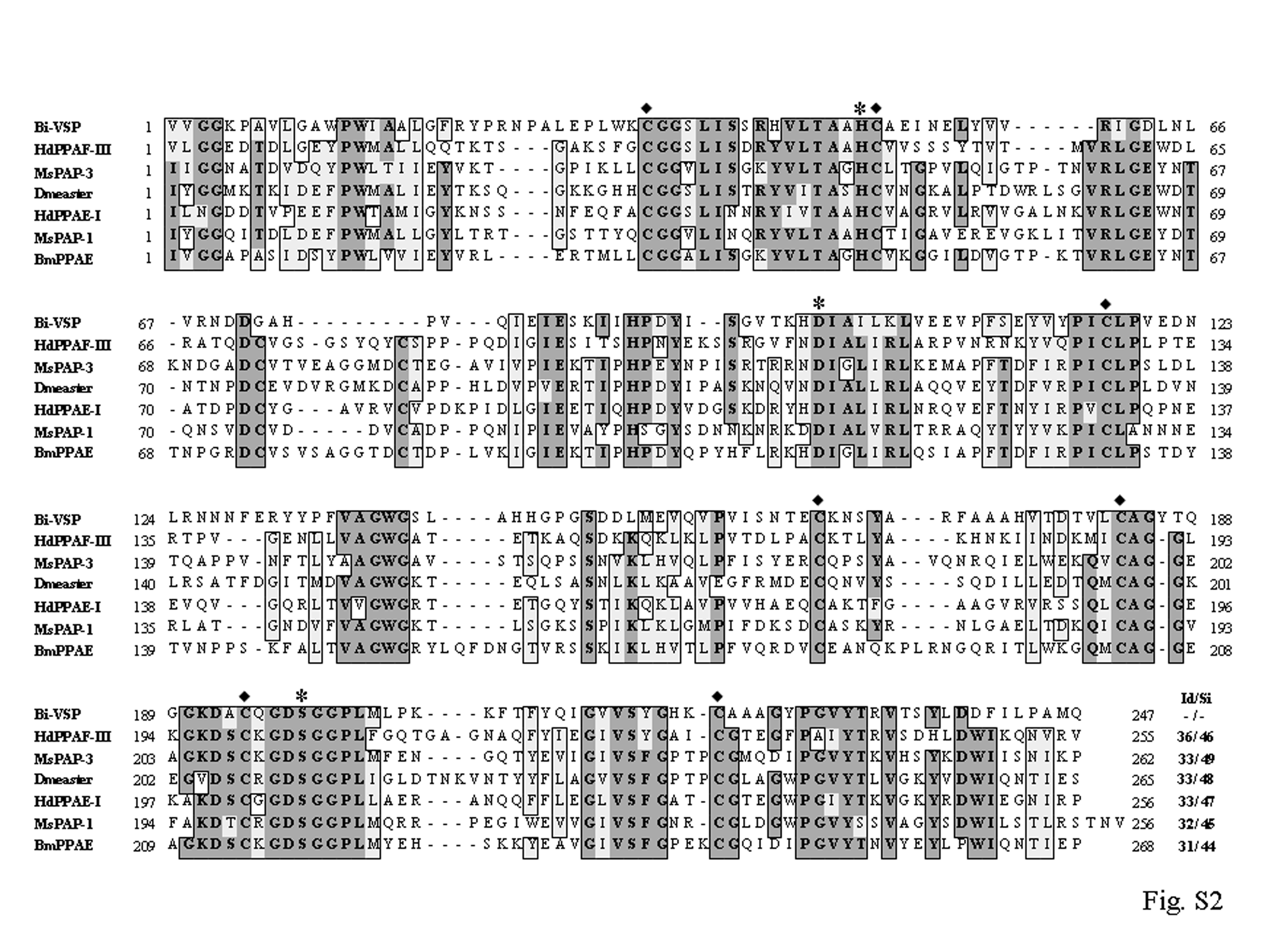

Supplement: Figure S2 — Amino acid sequence alignment of Bi-VSP with known PPAFs. Identical residues are shown in solid boxes. The dashes represent gaps that were introduced to preserve the alignment. The conserved cysteine residues in the SP domain are marked (solid squares), and the residues in the catalytic triad of the SP domain [His (H), Asp (D), and Ser (S)] are indicated with asterisks. The abbreviations and GenBank accession numbers for the aligned sequences are: Bi-VSP (this study, FJ159443); HdPPAF-III, Holotrichia diomphalia PPAF-III (BAC15604); MsPAP-3, Manduca sexta PAP-3 (AAX18637); Dmeaster, D. melanogaster easter (NP_524362); HdPPAE-I, H. diomphalia PPAE-I (BAA34642); MsPAP-1, M. sexta PAP-1 (AAX18636); and BmPPAE, B. mori PPAE (NP_001036832). The Bi-VSP sequence was used as a reference for the identity/similarity (Id/Si) values. (3.79 MB TIF) [file pone.0010393.s002.tif]

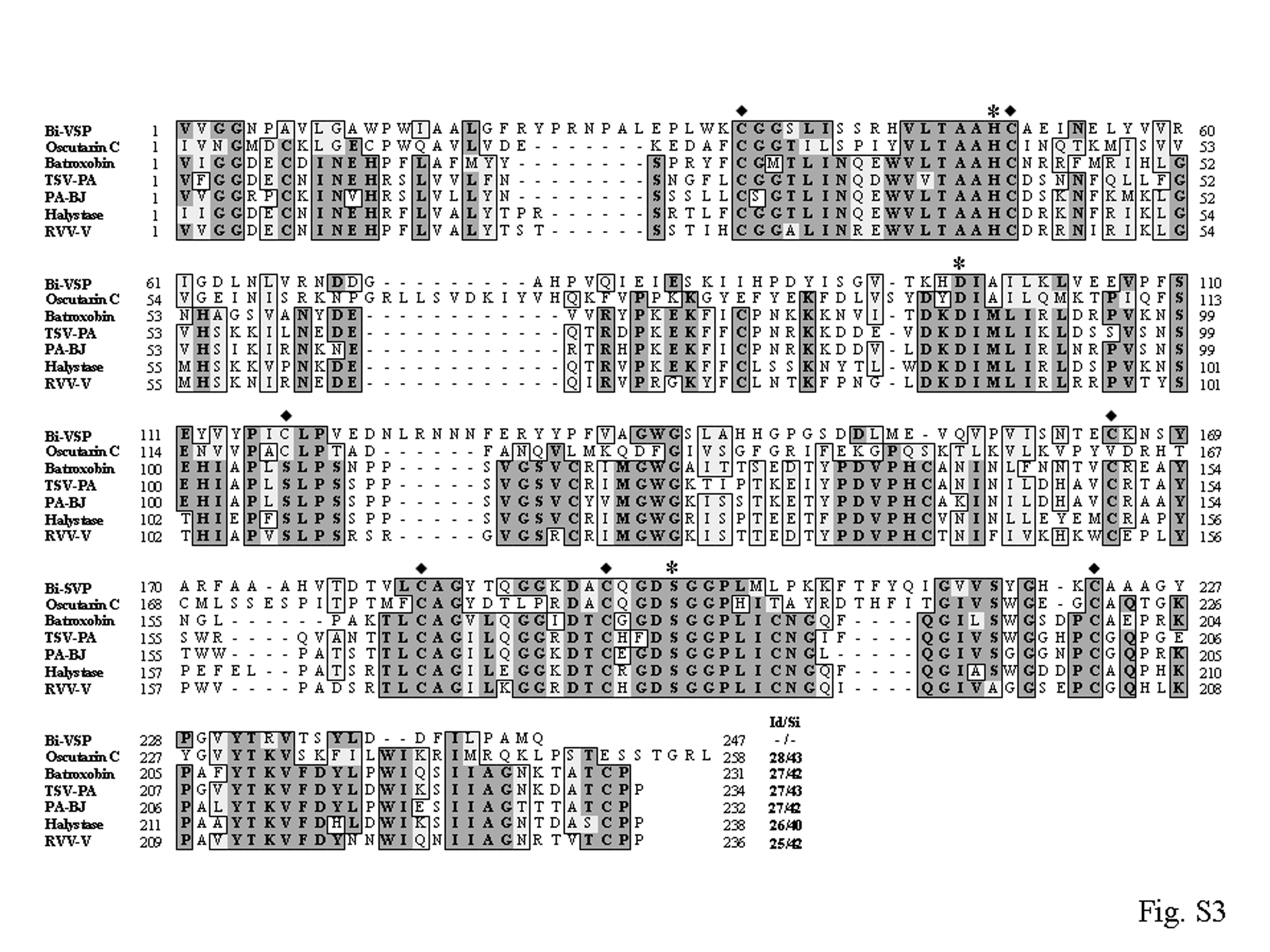

Supplement: Figure S3 — Amino acid sequence alignment of Bi-VSP with known snake venom serine proteases. Identical residues are shown in solid boxes. The dashes represent gaps that were introduced to preserve the alignment. The conserved cysteine residues in the SP domain are marked (solid squares), and the residues in the catalytic triad of the SP domain [His (H), Asp (D), and Ser (S)] are indicated with asterisks. The abbreviations and GenBank accession numbers for the aligned sequences are: Bi-VSP (this study, FJ159443), Oscutarin C (AY940204), Batroxobin (AAA48553), TSV-PA (Q91516), PA-BJ (P81824), Halystase (P81176), and RVV-V (P18964). The Bi-VSP sequence was used as a reference for the identity/similarity (Id/Si) values. (3.74 MB TIF) [file pone.0010393.s003.tif]

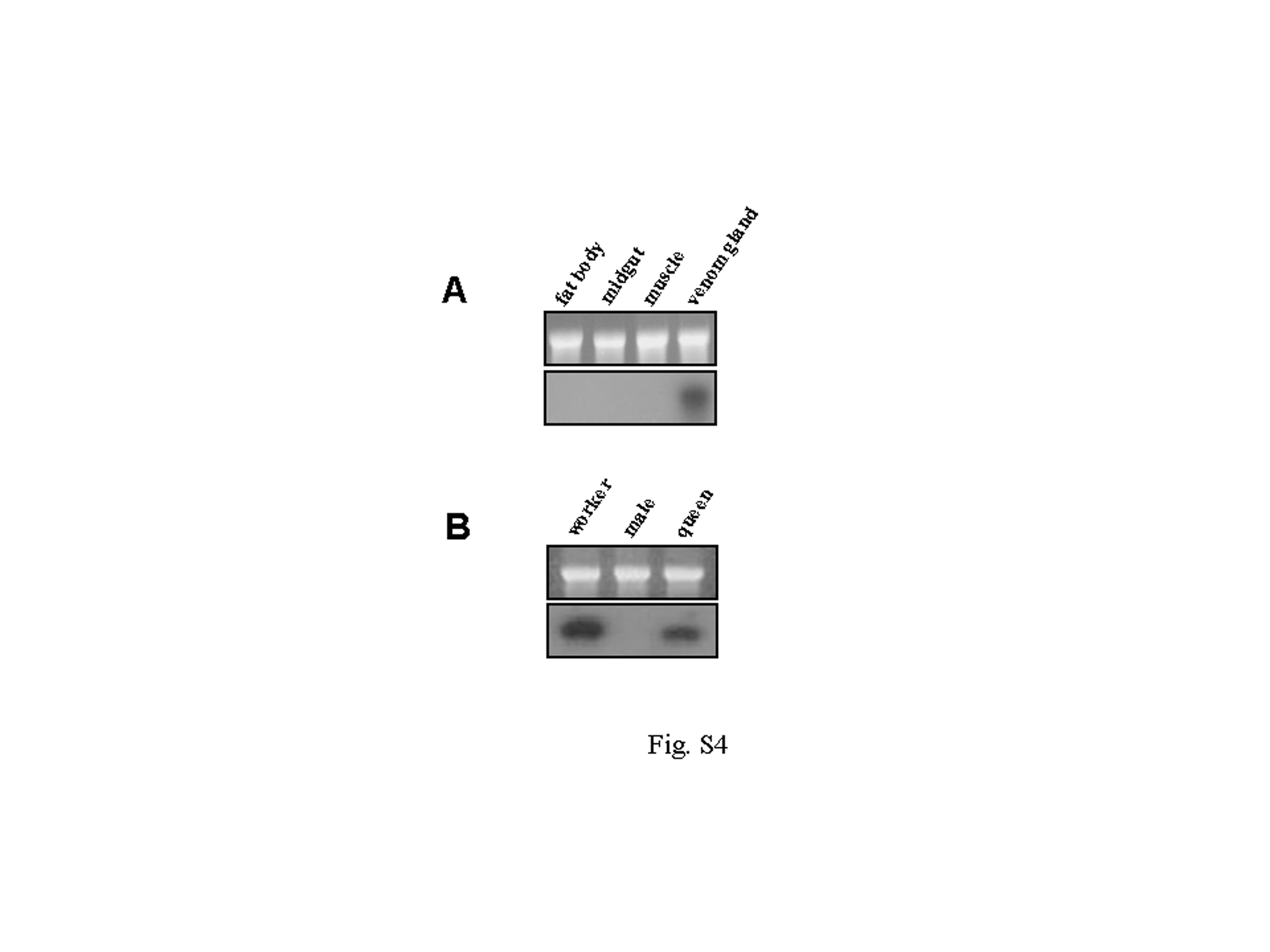

Supplement: Figure S4 — Northern blot analysis of Bi-VSP. (A) A northern blot for Bi-VSP was performed using total RNA isolated from the fat bodies, midgut, muscle, venom glands, and venom sacs of B. ignitus worker bees. (Top) Ethidium bromide staining of the RNA is shown to indicate equal loading. (Bottom) Bi-VSP transcripts. (B) A northern blot for Bi-VSP was performed using total RNA isolated from whole bodies of worker, male, and queen bees. (Top) Ethidium bromide staining of the RNA is shown to indicate equal loading. (Bottom) The Bi-VSP signal was present as a single band for the worker and queen, but not for the male, which lacks a sting apparatus. (0.68 MB TIF) [file pone.0010393.s004.tif]

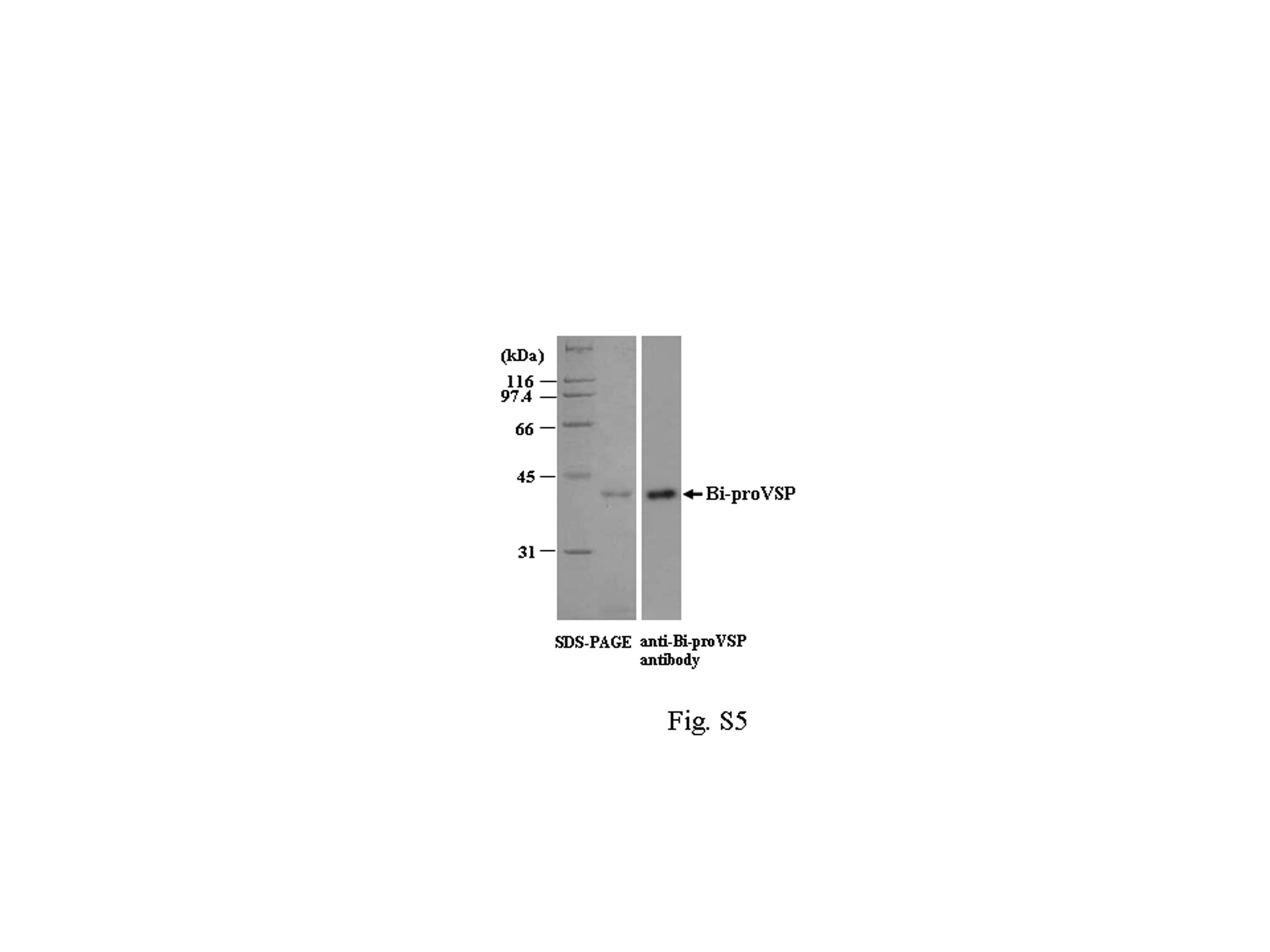

Supplement: Figure S5 — Purified recombinant Bi-proVSP. SDS-PAGE (left) and western blot (right) of recombinant Bi-proVSP purified from baculovirus-infected insect cells. The anti-Bi-proVSP antibody was produced in mice injected with recombinant Bi-proVSP. (0.64 MB TIF) [file pone.0010393.s005.tif]

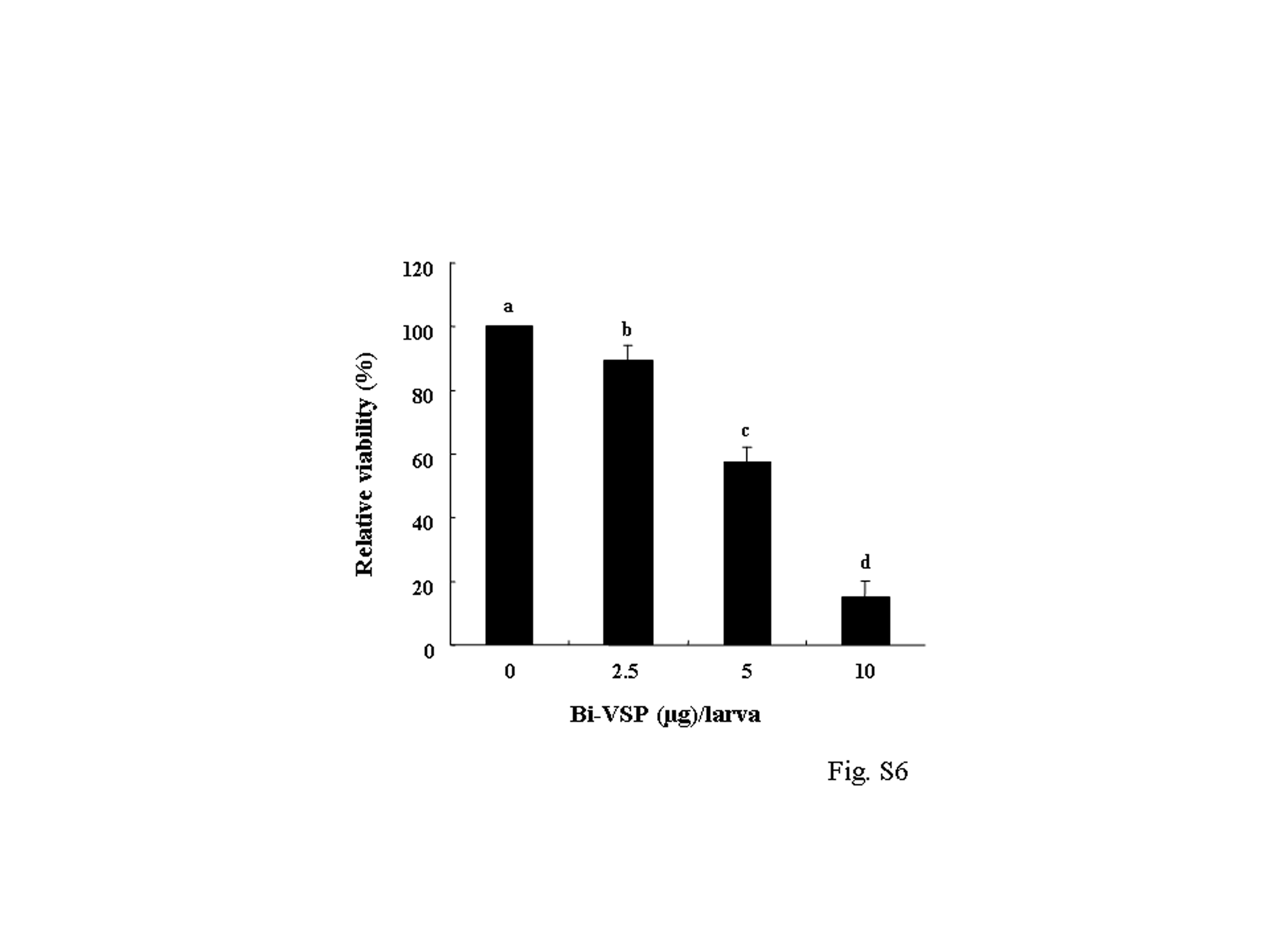

Supplement: Figure S6 — Viability of B. mori larvae injected with Bi-VSP. The viability of day 2 fifth-instar B. mori larvae injected with 1-10 µg of Bi-VSP per larva was surveyed at 24 h p.i. The data are expressed as the mean ± SD of assays performed in triplicate (n = 33). (0.62 MB TIF) [file pone.0010393.s006.tif]

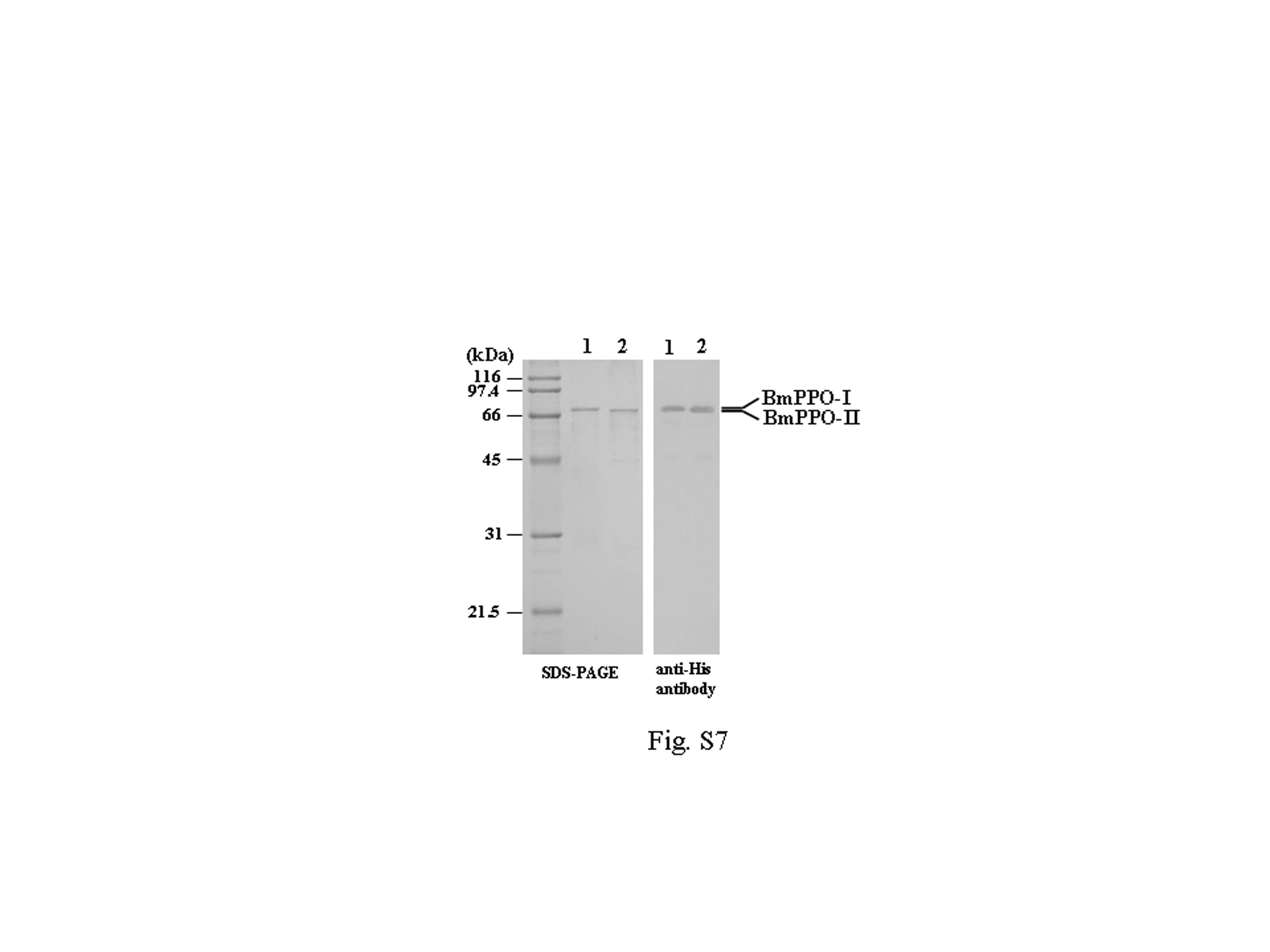

Supplement: Figure S7 — Purified recombinant B. mori proPOs expressed in baculovirus-infected insect Sf9 cells. SDS-PAGE (left) and western blot (right) of purified recombinant B. mori proPO-I (BmPPO-I) and proPO-II (BmPPO-II). His-tagged recombinant BmPPO-I and BmPPO-II were identified using an anti-His antibody. Lane 1, purified BmPPO-I; lane 2, purified BmPPO-II. (0.66 MB TIF) [file pone.0010393.s007.tif]

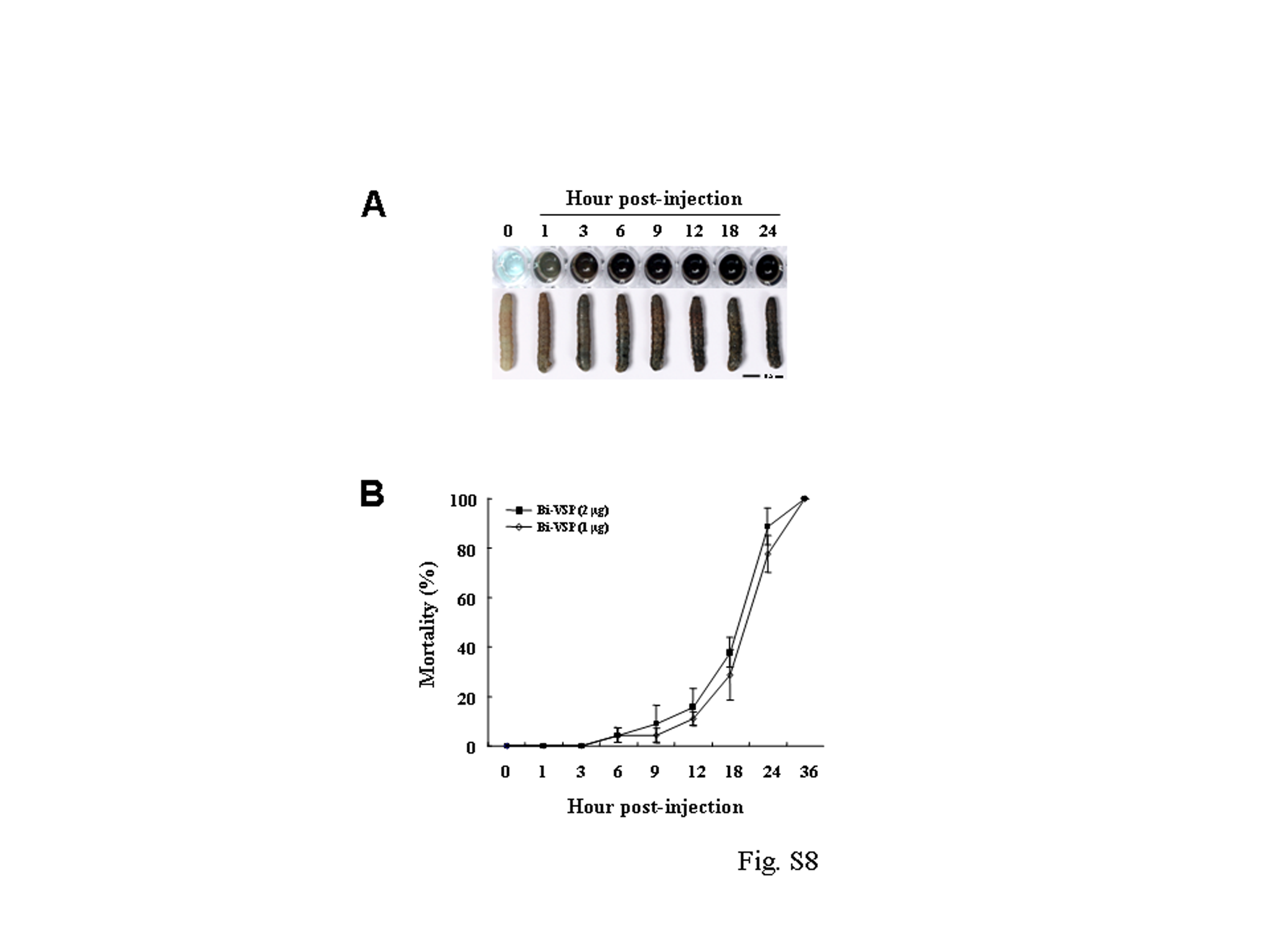

Supplement: Figure S8 — The mortality of S. exigua larvae injected with Bi-VSP. Day 2 fifth-instar S. exigua larvae were injected with Bi-VSP (1 µg or 2 µg per larva). (A) Fifth-instar S. exigua larvae and the collected hemolymph were photographed at various time points p.i. Scale bar, 0.5 cm. (B) The accumulated mortality was surveyed at various time points p.i. The data are expressed as the mean ± SD of assays performed in triplicate (n = 33). (1.09 MB TIF) [file pone.0010393.s008.tif]

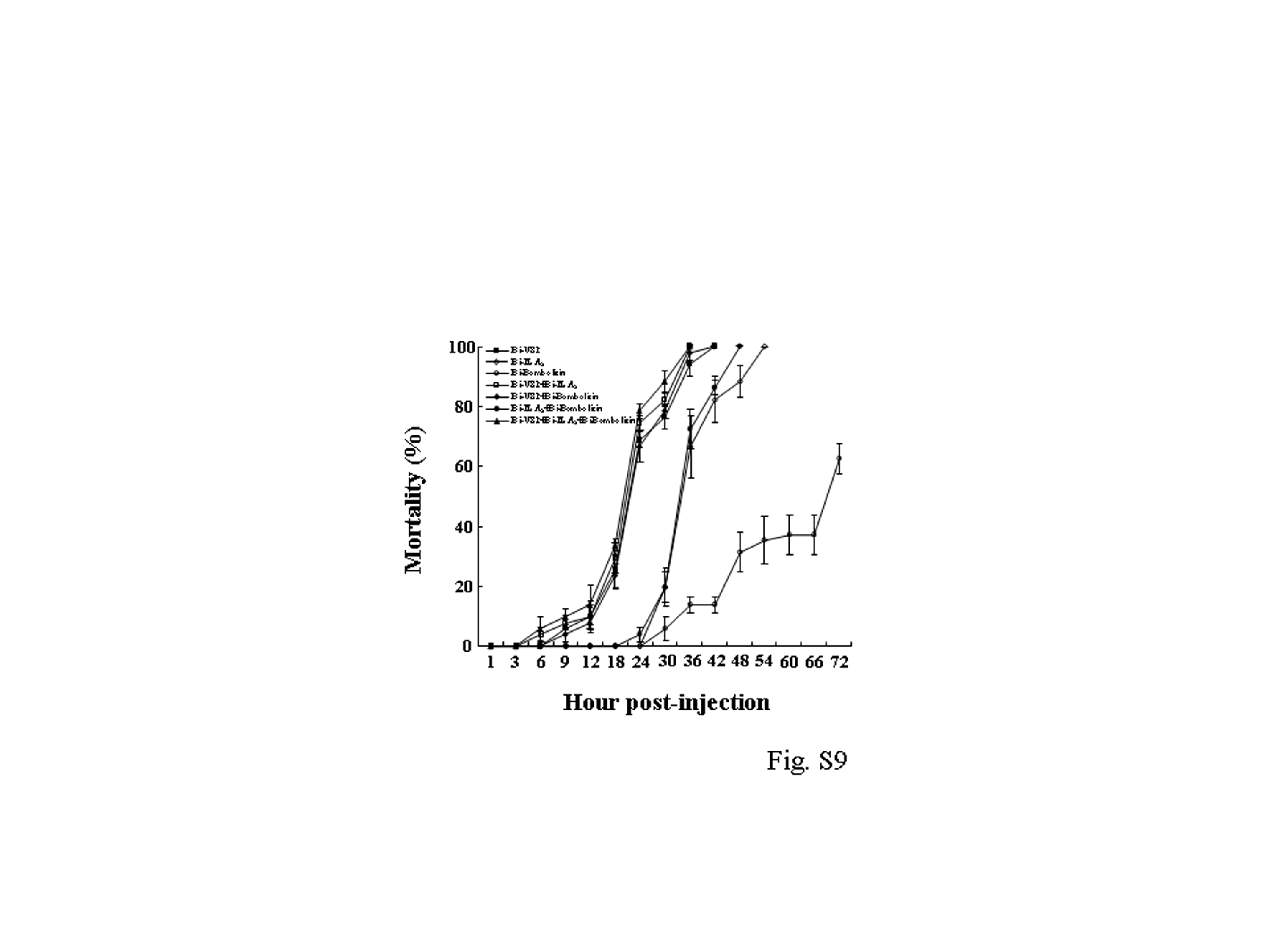

Supplement: Figure S9 — Mortality of Bi-VSP, Bi-PLA2, and Bi-Bombolitin in S. exigua larvae. Day 2 fifth-instar S. exigua larvae were injected with 2 µg/larva of Bi-VSP, Bi-PLA2, Bi-Bombolitin, Bi-VSP + Bi-PLA2, Bi-PAP + Bi-Bombolitin, Bi-PLA2 + Bi-Bombolitin, or Bi-VSP + Bi-PLA2 + Bi-Bombolitin. The data are expressed as the mean ± SD of three assays (n = 51). (0.78 MB TIF) [file pone.0010393.s009.tif]

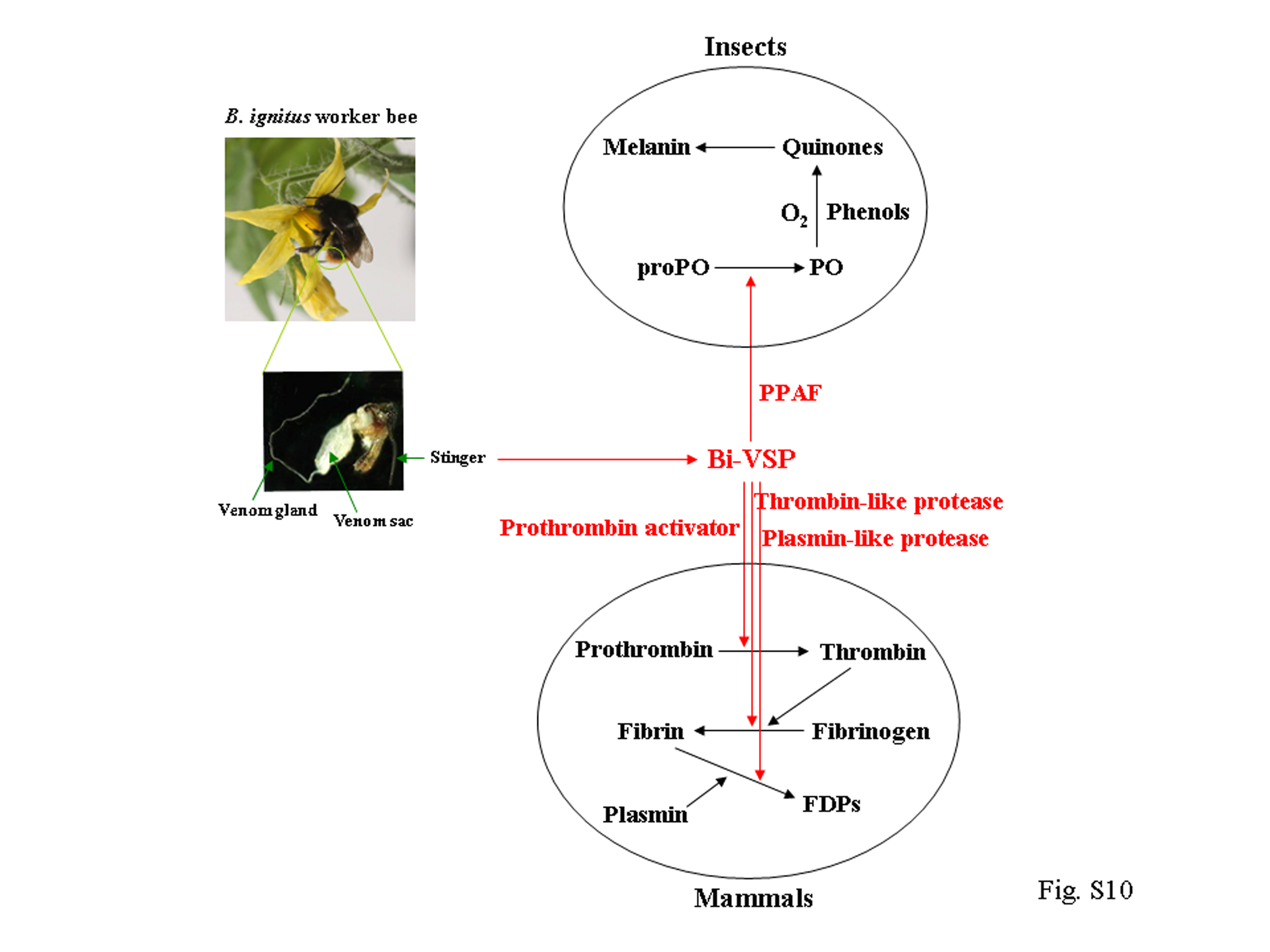

Supplement: Figure S10 — Proposed pathway for PO activation and the fibrin(ogen)olytic nature of Bi-VSP. The injection of bee venom via the sting apparatus results in the activation of proPO via Bi-VSP in the target insect. Elevated PO activity induces the death of target insects via melanization. In mammals, Bi-VSP not only activates prothrombin, it also degrades fibrinogen into fibrin degradation products (FDPs). (1.53 MB TIF) [file pone.0010393.s010.tif]
